# P-601. AI Enhanced Complication Prediction in Atypical Pneumonia: Validation of Predictive Clinical Parameters In a Pilot Study

**DOI:** 10.1093/ofid/ofaf695.814

**Published:** 2026-01-11

**Authors:** Adebanke Adeyemi, Swapan Nath

**Affiliations:** TCU School of Medicine, Fort Worth, Texas; TCU Burnett School of Medicine, Fort Worth, Texas

## Abstract

**Background:**

*Atypical pneumonia*, particularly that caused by *Mycoplasma pneumoniae*, presents unique diagnostic challenges due to its variable clinical course and potential for severe complications such as necrotizing pneumonia. Early identification of predictive features for disease progression is critical. We sought to evaluate the clinical, laboratory, and radiographic markers associated with necrotizing *Mycoplasma pneumoniae* pneumonia and assess the potential for artificial intelligence (AI) models to predict complications at initial presentation.

**Methods:**

We reviewed recent literature characterizing severe and necrotizing *Mycoplasma pneumonia* infections. We compiled key predictive features, including clinical presentation, radiologic findings, laboratory markers, and risk factors for complications. An AI-based framework was conceptualized utilizing these features to predict progression to necrotizing disease.

**Results:**

Clinical predictors included subacute onset of fever, dry cough, extrapulmonary manifestations, and rapid respiratory decline. Radiographic findings associated with complicated disease included segmental or lobar consolidation, ground-glass opacities, and early cavitation. Laboratory predictors encompassed elevated CRP, ESR, LDH, cold agglutinin positivity, and rising D-dimer levels. Risk factors for complications included delayed initiation of appropriate antibiotics, presence of bacterial co-infection, and hyperimmune responses. AI models incorporating early imaging findings, inflammatory marker kinetics, and time to appropriate therapy initiation demonstrate potential for early stratification of patients at risk for necrotizing progression.

**Conclusion:**

Identifying clinical, laboratory, and imaging predictors is essential for timely diagnosis and intervention in atypical pneumonia complicated by necrotizing *Mycoplasma pneumoniae*. AI-driven predictive models show promise in aiding early recognition of severe disease, ultimately improving patient outcomes. Prospective validation of these AI tools is warranted.

**Disclosures:**

All Authors: No reported disclosures

